# Rural–urban differences in smoking quit ratios and cessation-related factors: Results from a nationally representative sample

**DOI:** 10.1111/jrh.12870

**Published:** 2024-08-19

**Authors:** Devon Noonan, Suzanne Frisbee, Lorna Bittencourt, Dana Rubenstein, F. Joseph McClernon, Dana Mowls Carroll

**Affiliations:** 1 Duke University School of Nursing, Durham, North Carolina, USA; 2 Duke Cancer Institute, Durham, North Carolina, USA; 3 Division of Environmental Health Sciences, School of Public Health, University of Minnesota, Minneapolis, Minnesota, USA; 4 Department of Psychiatry and Behavioral Sciences, Duke University School of Medicine, Durham, North Carolina, USA; 5 Clinical and Translational Science Institute, Duke University School of Medicine, Durham, North Carolina, USA; 6 Masonic Cancer Center, University of Minnesota, Minneapolis, Minnesota, USA

**Keywords:** cessation, health equity, rural, tobacco use

## Abstract

**Purpose:**

There are significant rural/urban disparities that exist in cancer and chronic disease morbidity and mortality, many of which are attributed to increased tobacco use prevalence in rural populations compared to urban. Understanding differences in rural and urban tobacco use patterns is key to developing targeted interventions.

**Methods:**

Using nationally representative data from Wave 5 of the Population Assessment of Tobacco Use and Health (PATH), we examined weighted frequencies and conducted multivariable logistic regression to examine the use of cessation supports in people who currently smoke with a quit attempt in the last 12 months (recent attempters) by rural and urban status and geographic region. Our second objective was to examine lifetime quitting in rural versus urban people who smoke and by geographic region.

**Results:**

Rural people who recently attempted to quit were less likely to use any FDA- approved cessation aids, less likely to use Nicoctine Replacement Therapy (NRT), and less likely to be exposed to a home smoking ban in the adjusted analysis. The adjusted odds of quitting were lower in the rural Northeast, Midwest, and South compared to the urban regions.

**Conclusions:**

Findings from this data can serve to inform the development of targeted interventions for rural communities.

## INTRODUCTION

The rate of cigarette use is substantially greater in rural areas (19%) compared to the general population (11%).^1,2^ These higher rates of tobacco use in rural areas contribute to a higher risk of overall cancer mortality and a higher incidence of tobacco-related cancers in rural populations compared to nonrural.^3^ Reasons for differing tobacco use rates between rural and nonrural populations are multifaceted.^4,5^ Higher rates of smoking in rural areas are due in part to people in rural areas being less likely to make a quit attempt and having lower odds of cessation.^2^ There are documented disparities in smokefree policies, household smoking bans, and tobacco-related taxes in rural compared to urban areas that may contribute to certain beliefs and norms that contribute to tobacco use.^5–7^ The tobacco industry specifically targets rural communities, and it is documented that rural areas have less access to cessation services and health care providers, a structural barrier to cessation.^2^

Providing updated estimates on differences in the use of cessation supports and quitting between urban and rural populations is a critical step in the tobacco control movement so that public health efforts can be shaped to better meet the unique cessation needs of rural populations.^8^ To date, however, there is little evidence on a national scale that has examined differences in cessation factors and quit ratios between rural and urban populations. Even less work has focused on regional variations in cessation-related outcomes among rural people.^9,10^ To address this lack of knowledge and expand past literature in this area, our first objective was to examine the prevalence of cessation supports, including both medicinal and behavioral, among those who currently smoke with a past year quit attempt (recently attempted to quit) by rural and urban status and geographic region. Odds of cessation are significantly higher among those who use cessation supports,^11^ so understanding differences in cessation support among rural/urban status can provide information for tailoring interventions to increase the use of cessation aids. Our second objective was to examine lifetime quitting in rural versus urban people who smoke and by geographic region. We used data from Wave 5 of the Population Assessment of Tobacco Use and Health (PATH), a large, nationally representative data set. Findings from this study can serve to inform the development of targeted interventions for rural communities.

## METHODS

### Data

We used PATH Wave 5 data (from December 2018 to November 2019) for the current analysis. The PATH study is a nationally representative longitudinal cohort study that surveys tobacco use patterns and their associated effects on health outcomes of US youth and adults aged 12 and older. Additional details on the PATH study design and methods are published and described in detail elsewhere.^12^ This secondary data analysis was deemed not needed to be approved by the Institutional Review Board at the University of Minnesota.

### Sample and measures

We determined the rural and urban status of participants using geographical units called segments, which were based on US Census blocks and the US Census Bureau’s definition of rural versus urban. The PATH sampling procedure identified a segment as “urban” if the majority of its total population resided in areas classified as urban according to the 2010 census (i.e., a minimum population density of at least 25,000 people). Otherwise, segments were “not urban,” which we have referred to as “rural” as done in a prior study.^13^ Only the Wave 1 (2012–2013) data set included this rural measure, and as a result, only participants in Wave 5 who also participated in Wave 1 were included in our analyses and their Wave 1 rural or urban status was assumed to be their Wave 5 status. We also determined regionality based on census region. PATH grouped the 50 states and the District of Columbia into four regions: Northeast, Midwest, South, and West. To examine cessation-related supports, we restricted the data to adult respondents who reported current daily smoking (i.e., reported ever smoking a cigarette, having smoked more than 100 cigarettes in their lifetime, and currently smoking every day or someday smoking) and reported a quit attempt in the past 12 months (recently attempted quitting). Lifetime quitting estimates were operationalized by the number of people who reported former smoking (defined as smoking 100 cigarettes in their lifetime and not currently smoking) divided by the number of people who ever smoked (defined as smoking at least 100 cigarettes in their lifetime). Characteristics of participants, potential confounders, and independent variables (cessation supports) are detailed in Table 1

### Statistical analyses

Analyses were conducted in SAS Version 9.4 (SAS Institute, Inc) using survey procedures and *Wave 5: Adult-Wave 1 Cohort All-Waves Weights*. Variances and 95% confidence intervals (CIs) of the estimates were calculated by the balanced repeated replication method with Fay’s adjustment set to 0.3 to increase stability to account for the PATH study design.^14^

For cessation-related factors, chi-square tests for association were used to produce prevalence estimates and 95% CIs followed by unadjusted and adjusted analyses using multivariable logistic regression accounting for the potential confounders. Cessation-related factors between rural and urban were examined overall and within each geographic region, except the West since the sample size of rural participants was limited (*n* = 40). While the population of focus for use of cessation-related factors is people who attempted to quit but remained smoking, sensitivity analyses were conducted among the following two populations: (1) people who attempted to quit but remained smoking *and* people who attempted to quit and succeeded and (2) people who attempted to quit and succeeded only.

Quit ratios were also examined via unadjusted analyses and via multivariable logistic regression including the potential confounders such as age, gender, race/ethnicity, and educational attainment. Two-sided *p*-values <0.05 were used to detect statistically significant differences between rural and urban People Who Smoke (PWS). Quit ratios between rural and urban were examined overall and within each geographic region.

## RESULTS

### Participant characteristics

When compared with their urban counterparts, rural people who smoke with a past year quit attempt were more likely to be White non-Hispanic race/ethnicity, have lower educational attainment, have greater cigarette dependence (based on average Cigarettes per Day (CPD) and time to first cigarette), and have lower quit interest.

### Cessation-related factors among people who recently attempted quitting

As shown in Table 1, rural versus urban people who smoke with a past year quit attempt did not differ in terms of prevalence estimates (or unadjusted odd ratios [ORs]) of cessation supports with the exception of a household smoking ban. Specifically, 55.49% of rural versus 63.93% of urban people who smoke with a past year quit attempt had a household smoking ban (unadjusted odds: 0.70; *p* = 0.0006; Table 2)— a finding that is likely driven by the South census region (unadjusted odds: 0.56; *p* ≤ 0.0001; Table 2). In adjusted analyses, the association with a household smoking ban was upheld for the South region. Specifically, in the South region, the adjusted ORs of having a household smoking ban were 27% lower in rural versus urban people who smoke with a past year quit attempt (*p* = 0.0450).

Adjusted analyses revealed differences in medication and ecigarette use that were not found in unadjusted analyses. Specifically, the adjusted ORs for use of cessation medication (NRT, prescriptions) were 23% lower among rural versus urban people who smoke (*p* = 0.0382). Likewise, the adjusted odds of using an e-cigarette to help quit smoking were 30% lower among rural versus urban people who smoke (*p* = 0.0354). The discordant findings between unadjusted and adjusted analyses for these two outcomes appear to be due to confounding effects (Table S1). For example, the likelihood of using cessation medication is independently associated with age, gender, race, education, cigarette dependence, and quit interest, which are also all associated with rurality.

Sensitivity analyses revealed similar results for all cessation- related measures with the exception of medication and e-cigarette use (Table S2). In the analysis restricted to people who succeeded in quitting, opposite findings were observed for medication and e-cigarette use from the primary sample of those who attempted to quit but failed. For example, rural versus urban succeeders had greater adjusted odds of using medications (*p* = 0.0396) for quitting smoking.

### Quit ratios

Among people who reported ever smoking (unweighted *n* = 13,082), 56% of rural and 61% of urban report lifetime quitting (*p* = 0.0014). The adjusted odds of lifetime quitting are reported at the bottom of Table 2 and are 23% lower among rural versus urban respondents (*p*< 0.0001). The adjusted odds of lifetime quitting by region were also significantly lower in rural versus urban areas for the Northeast, Midwest, and South regions.

## DISCUSSION

Rural disparities exist in smoking and smoking-attributed cancers in the United States.^15^ Our findings support this continued tobacco use disparity between rural and urban populations. We found lower adjusted odds of medication use to aid in quitting in rural versus urban people who smoke. In the Southern region only, those of rural residence were less likely to have a household smoking ban compared to their urban counterparts. We also found lower quit ratios in rural compared to urban and regional differences in quit ratios.

Prior work supports that rural people smoke at a higher rate and are less likely to make a quit attempt, compared to urban populations.^2^ Further, heaviness of smoking and nicotine dependence are associated with decreased odds of cessation.^16,17^ Our finding that rurality is associated with cessation medication use, and specifically a lower odds compared to urban when adjusting for covariates, is significant—a difference that was not observed in unadjusted analyses. An interpretation of these findings is that when rural and urban participants are made more “similar” in terms of multiple social determinants of health (e.g., race, education) and cigarette dependence, rurality emerges as an independent risk factor for lower medication use. A similar interpretation can be made for using an e-cigarette to support quitting. These findings highlight the role of intersectionality in rural health and thus an important consideration when designing and evaluating interventions.

Significant barriers exist for many rural populations in access to cessation supports, and this may account for the disparities in medication use. Compounded with the fact that provider shortages are also common in rural areas and rural populations must travel far distances for care, there is a need to leverage telehealth and other remote approaches (e.g., text-based interventions) to increase access to evidence-based cessation support.^18,19^ Remote approaches need to be combined with interventions that address the structural issues that perpetrate these barriers, including policy changes that incentivize rural workforce development and increase public health funding and infrastructure to support on-the-ground cessation services.

It is noteworthy that we found differences in household smoking bans when adjusting for covariates between rural and urban people who smoke in the Southern region. Indoor smoking bans reduce smoking and encourage abstinence,^20^ and recent research suggests that among those actively trying to quit, implementing any type of home smoking ban while quitting significantly increases the likelihood of cessation.^21^ Significantly lower prevalence of household smoking bans in rural areas compared to urban, as we found, may contextualize some of our findings including lower quit ratios in rural versus urban people.

Another notable finding is that, in sensitivity analyses, we observed a greater odds of medication use among those who attempted to quit and succeeded if they were rural residing versus urban residing—an opposite finding from when the sample was restricted to those who attempted to quit but failed. One interpretation of these results is that for rural succeeders (who tend to have more exposure to positive social norms around smoking than urban succeeders), medication use during a quit attempt is more critical to quitting success than urban suc- ceeders. Thus, this data point supports the need for efforts that help increase medication use in rural people who smoke. This finding also suggests that rural succeeders are inherently different from rural people who tried to quit but failed. For example, rural succeeders were younger on average and potentially more likely to be open to and seek out medication use and e-cigarettes to quit. Further research on these intersectional factors is recommended.

We also found regional variations in quit ratios between rural and urban people with lower quit ratios in the rural Northeast, Midwest, and South. This supports prior work that highlights regional differences in tobacco use and within region differences between rural and urban populations, particularly in the Northeast and the South.^10^ These regional differences highlight the heterogeneity of rural populations^10^ and underscore the need for targeted cessation efforts in specific geographic areas with high prevalence rates to reduce tobacco-related health disparities.

This study is not without limitations. Survey data from the PATH study are based on self-report and are thus subject to recall bias. Our regional analyses are likely underpowered; however, they provide signals that can guide future work in this area. We were limited to the measures provided in PATH, for example, cessation aid use was only reported for the most recent quit attempt, when people could have used medications for prior attempts that year. We also assumed the rural status of participants at baseline (2012–2013) reflects their rural status at Wave 5 (2018–2019) as rural status was not reassessed at any follow-up wave in PATH. Finally, data restrictions prevented geographic analyses at state and/or county levels.

## CONCLUSIONS

In conclusion, rural populations continue to smoke at high rates and have lower quit ratios compared to their urban counterparts. Our findings further underscore the need for targeted smoking cessation interventions and resources in rural communities, with particular attention to leveraging rural strengths that can potentially translate into protective factors and catalysts toward cessation. A multilevel approach is needed to provide not only individual-level cessation support but also the enactment of policies that address persistent structural barriers (e.g., lack of health care providers, access and accessibility of cessation support, and lack of indoor/home smoking bans) to tobacco use disparities in rural communities.

## Supplementary Material

Supp Table 1

Supp Table 2

Additional supporting information can be found online in the Supporting Information section at the end of this article.

## Figures and Tables

**Table 1.** Characteristics of rural and urban people who smoke with a past year quit attempt and prevalence estimates of cessation supports and lifetime quitting

	Rural (n=577; n_w_=2,956,888)	Urban(n=1,798; n_w_=9,572,050)	*p-value*
*Socio-demographics and smoking behavior among PWS with a past year quit attempt*			
Age in years	46.28 (44.86, 47.69)	44.64 (43.84, 45.55)	0.0519
Female biological sex	51.98 (46.89, 57.07)	48.31 (45.80, 50.83)	0.2081
White, non-Hispanic race/ethnicity	81.90 (76.35, 87.45)	57.58 (54.42, 60.74)	**<.0001**
Education greater than high school	41.69 (36.70, 46.68)	47.65 (44.58, 50.72)	**0.0389**
Cigarettes per day	13.13 (12.72, 14.18)	10.02 (9.49, 10.54)	**<.0001**
Time to first cigarette (< 30 minutes)	63.52 (58.38, 68.66)	50.92 (47.92, 53.92)	**<.0001**
Quit interest (scale 1 to 10)	8.05 (7.90, 8.20)	7.75 (7.53, 7.96)	**0.0364**
Past 30 day e-cigarette use	23.96 (20.01, 28.41)	24.95 (22.47, 27.61)	0.6870
Past 30 day smokeless tobacco use	4.62 (3.19, 6.65)	3.34 (2.52, 4.43)	0.1314

*Cessation supports among PWS with a past year quit attempt*			
Advised by a doctor in past 12 months to quit (Yes)	52.03 (46.65, 57.41)	49.22 (46.44, 52.00)	0.3789
Smoking ban in the home (Yes)	55.49 (51.20, 59.78)	63.93 (61.20, 66.65)	**0.0003**
Social support (Yes)	30.86 (27.08, 34.63)	32.64 (29.71, 35.56)	0.4582
Behavioral support (Yes)	9.71 (6.72, 12.71)	8.88 (7.29, 10.47)	0.5889
FDA approved cessation medication (Yes)	27.42 (23.23, 31.61)	27.76 (25.29, 30.22)	0.8864
NRT (Yes)	19.40 (16.30, 22.50)	20.55 (18.32, 22.77)	0.5560
Prescription med (Yes)	10.86 (7.83, 13.89)	10.66 (9.03, 12.29)	0.9045
E-cigarette to help quit (Yes)	13.50 (9.60, 17.40)	14.75 (12.76, 16.75)	0.5565
SLT to help quit (Yes)	0.94 (0.20, 1.68)	1.75 (0.32, 2.57)	0.1993

*Among ever smokers*			
Lifetime quitting	56.64 (53.89, 59.42)	61.42 (60.16, 62.67)	**0.0014**

n_w_ reflects weighted sample size

**Table 2.** Odds of cessation methods/supports and lifetime quitting among rural versus urban PWS.

Odds of cessation methods/supports among rural vs urban PWS with a past quit attempt	All U.S. (N_r_=577; N_u_=1,798)	*p-value*	Northeast region (N_r_=99; N_u_=236)	*p-value*	Midwest region (N_r_=157; N_u_=499)	*p-value*	South region (N_r_=281; N_u_=659)	*p-value*	West region (N_r_=40; N_u_=404)	*p-value*
**Advised by a doctor to quit**										
Unadjusted	1.12 (0.87, 1.45)	0.3880	1.40 (0.74, 2.66)	0.3035	0.91 (0.55, 1.50)	0.7050	1.22 (0.84, 1.77)	0.2926	0.79 (0.55, 1.13)	0.1986
Adjusted^a^	1.01 (0.78, 1.32)	0.9334	1.56 (0.70, 3.51)	0.2767	0.94 (0.56, 1.57)	0.7998	1.11 (0.80, 1.53)	0.5327	-	

**Smoking ban in the home**										
Unadjusted	0.70 (0.58, 0.86)	**0.0006**	0.81 (0.47, 1.39)	0.4337	1.15 (0.70, 1.88)	0.5797	0.56 (0.43, 0.73)	**<.0001**	1.32 (0.45, 3.88)	0.6055
Adjusted^a^	0.87 (0.69, 1.10)	0.2542	0.97 (0.45, 2.06)	0.9309	1.13 (0.64, 2.02)	0.6697	0.73 (0.54, 0.99)	**0.0450**	-	

**Social support**										
Unadjusted	0.92 (0.74, 1.15)	0.4616	1.19 (0.72, 1.99)	0.4983	0.69 (0.43, 1.12)	0.1346	1.01 (0.73, 1.39)	0.9703	0.83 (0.45, 1.55)	0.5635
Adjusted^a^	0.91 (0.72, 1.16)	0.4410	1.03 (0.56, 1.90)	0.9143	0.66 (0.41, 1.08)	0.0972	1.06 (0.74, 1.53)	0.7316	-^b^	

**Behavioral support**										
Unadjusted	1.10 (0.76, 1.61)	0.6039	1.94 (0.68, 5.54)	0.2156	0.89 (0.45, 1.78)	0.7370	1.10 (0.69, 1.74)	0.6864	-^b^	
Adjusted^a^	0.98 (0.69, 1.39)	0.9049	1.33 (0.37, 4.75)	0.6560	1.01 (0.53, 1.92)	0.9674	1.03 (0.63, 1.67)	0.9135	-^b^	

**FDA approved cessation med**										
Unadjusted	0.98 (0.78, 1.25)	0.8886	0.87 (0.58, 1.29)	0.4720	0.75 (0.44, 1.30)	0.3053	1.27 (0.86, 1.87)	0.2235	0.98 (0.54, 1.81)	0.9568
Adjusted^a^	0.77 (0.60, 0.99)	**0.0382**	0.67 (0.41, 1.12)	0.1295	0.62 (0.35, 1.13)	0.1187	1.06 (0.69, 1.61)	0.7960	-^b^	

**E-cigarette**										
Unadjusted	0.90 (0.63, 1.29)	0.5649	0.51 (0.21, 1.23)	0.1311	0.81 (0.42, 1.57)		1.04 (0.60, 1.81)	0.8756	1.48 (0.41, 5.35)	0.5431
Adjusted^a^	0.70 (0.50, 0.98)	**0.0354**	0.40 (0.14, 1.19)	0.1001	0.67 (0.33, 1.35)	0.3427	0.72 (0.46, 1.13)	0.1522	-^b^	
Odds of lifetime quitting among rural vs urban persons who have ever smoked	All U.S. (N_r_=3,178; N_u_=9,904)	*p-value*	Northeast region (N_r_=546; N_u_=1,389)	*p-value*	Midwest region (N_r_=816; N_u_=2,614)	*p-value*	South region (N_r_=1,524; N_u_=3,482)	*p-value*	West region (N_r_=292; N_u_=2419)	*p-value*

Unadjusted	0.82 (0.72, 0.93)	0.0025	0.82 (0.67, 0.99)	0.0449	0.88 (0.71, 1.09)	0.2423	0.81 (0.66, 0.98)	0.0304	1.18 (0.82, 1.70)	0.3805
Adjusted^b^	0.77 (0.68, 0.87)	**<.0001**	0.72 (0.56, 0.93)	**0.0130**	0.78 (0.64, 0.94)	**0.0093**	0.78 (0.64, 0.96)	**0.0176**	1.02 (0.79, 1.31)	0.8835

a : Adjusted for age, sex, race, education, time to first cigarette, CPD, and quit interest. b Adjusted for age, sex, race, education

b : Limited sample size (N=40) and adjusted results not included
