# Supplementary material for: Rural–urban differences in smoking quit ratios and cessation-related factors: Results from a nationally representative sample
Source: J Rural Health. Author manuscript; Available in PMC 2026 Apr 27. (PMC13112512; doi:10.1111/jrh.12870)
Supplement: Supp Table 1 [file NIHMS2164903-supplement-Supp_Table_1.docx]

| **Supplementary Table 1** | | |
| --- | --- | --- |
| **Odds of lifetime quitting**  (N_r_=3,178; N_u_=9,904) | **AOR** | **p-value** |
| Rural versus Urban | 0.77 (0.68, 0.87) | <.0001 |
| Age | 1.04 (1.04, 1.05) | <.0001 |
| Female versus Male | 0.87 (0.80, 0.95) | 0.0013 |
| White versus Other Race Groups | 1.24 (1.11, 1.38) | 0.0002 |
| Lower versus higher education | 0.41 (0.38, 0.45) | <.0001 |
| **Odds of FDA approved cessation medication use**  All U.S. (N_r_=577; n_w_=2,956,888) (N_u_=1,798; n_w_=9,572,050) | **AOR** | **p-value** |
| Rural versus Urban | 0.77 (0.60, 0.99) | 0.0382 |
| Age | 1.03 (1.02, 1.04) | <.0001 |
| Female versus Male | 1.30 (1.03, 1.65) | 0.0279 |
| White versus Other Race Groups | 1.31 (1.03, 1.67) | 0.0286 |
| Lower versus higher education | 0.75 (0.61, 0.92) | 0.0060 |
| CPD | 1.02 (1.00, 1.04) | 0.0541 |
| Quit interest | 1.07 (1.02, 1.13) | 0.0062 |
| Time to first cigarette < 30 versus 30 + | 1.81 (1.40, 2.35) | <.0001 |
| **Odds of e-cigarette use to help quit smoking**  All U.S. (N_r_=577; n_w_=2,956,888) (N_u_=1,798; n_w_=9,572,050) | **AOR** | **p-value** |
| Rural versus Urban | 0.70 (0.51, 0.97) | 0.0354 |
| Age | 0.97 (0.95, 0.98) | <.0001 |
| Female versus Male | 1.23 (0.94, 1.60) | 0.1347 |
| White versus Other Race Groups | 2.16 (1.56, 3.00) | <.0001 |
| Lower versus higher education | 0.75 (0.55, 1.02) | 0.0695 |
| CPD | 1.00 (0.98, 1.01) | 0.6579 |
| Quit interest | 1.13 (1.04, 1.22) | 0.0039 |
| Time to first cigarette < 30 versus 30 + | 1.39 (1.00, 1.94) | 0.0499 |
