# Supplementary material for: Rural–urban differences in smoking quit ratios and cessation-related factors: Results from a nationally representative sample
Source: J Rural Health. Author manuscript; Available in PMC 2026 Apr 27. (PMC13112512; doi:10.1111/jrh.12870)
Supplement: Supp Table 2 [file NIHMS2164903-supplement-Supp_Table_2.docx]

**Supplementary Table 2.**

|  | Adjusted odds of cessation methods among rural versus urban people who *both failed (still smoking) and succeeded* *at quitting* between Wave 4 and 5 | | Adjusted odds of cessation methods among rural versus urban people *who succeeded at quitting* between  Wave 4 and 5 | |
| --- | --- | --- | --- | --- |
|  | **AOR (95% CI)** | ***p-value*** | **AOR (95% CI)** | ***p-value*** |
| **Advised by a doctor to quit** | 1.05 (0.81, 1.35) | 0.7280 | 1.13 (0.62, 2.04) | 0.6927 |
| **Smoking ban in the home** | 0.86 (0.69, 1.07) | 0.1642 | 0.82 (0.42, 1.61) | 0.5650 |
| **Social support** | 0.94 (0.75, 1.18) | 0.5667 | 1.22 (0.66, 2.28) | 0.5218 |
| **Behavioral support** | 1.21 (0.85, 1.73) | 0.2947 | 1.49 (0.50, 4.47) | 0.4731 |
| **FDA approved cessation med** | 0.96 (0.75, 1.24) | 0.7675 | 1.84 (1.03, 3.28) | **0.0396** |
| **E-cigarette use** | 0.87 (0.64, 1.20) | 0.3948 | 2.25 (0.84, 6.07) | 0.1075 |

Adjusted for age, sex, race, education, time to first cigarette (Wave 4), CPD (Wave 4), and quit interest (Wave 4).
